# Possible misdiagnosis of HIV associated lymphoma as tuberculosis among patients attending Uganda Cancer Institute

**DOI:** 10.1186/s12981-017-0139-x

**Published:** 2017-03-14

**Authors:** Paul Buyego, Lydia Nakiyingi, Henry Ddungu, Stephen Walimbwa, Damalie Nalwanga, Steven J Reynolds, Rosalind Parkes-Ratanshi

**Affiliations:** 1 Department of Medicine, Makerere University College of Health Sciences, Kampala, Uganda; 2 Uganda Cancer Institute Mulago Hospital, Kampala, Uganda; 3 Infectious Disease Institute, Makerere University College of Health Sciences, P.O. Box 22418, Kampala, Uganda; 4 Division of Intramural Research, National Institute of Allergy and Infectious Diseases, National Institutes of Health, Bethesda, MD USA; 5 Johns Hopkins School of Medicine, Baltimore, MD USA; 6 University of Cambridge, Cambridge, UK

**Keywords:** Tuberculosis, Uganda, Lymphoma, HIV, Misdiagnosis

## Abstract

**Background:**

Early diagnosis of HIV associated lymphoma is challenging because the definitive diagnostic procedure of biopsy, requires skills and equipment that are not readily available. As a consequence, diagnosis may be delayed increasing the risk of mortality. We set out to determine the frequency and risk factors associated with the misdiagnosis of HIV associated lymphoma as tuberculosis (TB) among patients attending the Uganda Cancer Institute (UCI).

**Methods:**

A retrospective cohort study design was used among HIV patients with associated lymphoma patients attending the UCI, Kampala, Uganda between February and March 2015. Eligible patient charts were reviewed for information on TB treatment, socio-demographics, laboratory parameters (Hemoglobin, CD4cells count and lactate dehydrogenase) and clinical presentation using a semi structured data extraction form.

**Results:**

A total of 183 charts were reviewed; 106/183 were males (57.9%), the median age was 35 (IQR, 28–45). Fifty six (30.6%) patients had a possible misdiagnosis as TB and their median time on TB treatment was 3.5 (1–5.3) months. In multivariate analysis the presence of chest pain had an odd ratio (OR) of 4.4 (95% CI 1.89–10.58, p < 0.001) and stage III and IV lymphoma disease had an OR of 3.22 (95% CI 1.08–9.63, p < 0.037) for possible misdiagnosis of lymphoma as TB.

**Conclusion:**

A high proportion of patients with HIV associated lymphoma attending UCI are misdiagnosed and treated as TB. Chest pain and stage III and IV of lymphoma were associated with an increased risk of a possible misdiagnosis of lymphoma as TB.

## Background

Worldwide, 35 million people were living with HIV at the end of 2013 [1]. Sub-Saharan Africa remains the most severely affected region, accounting for 71% of the people living with HIV [1]. In Uganda at least 130,000 HIV new infections occur every year [2]. In 2013, an estimated 1.6 million Ugandans were living with HIV, and an estimated 63,000 people died of AIDS-related illnesses [3] including HIV related cancers. In the African setting Kaposi sarcoma remains the commonest AIDS related malignancy and lymphoma has also increased due to HIV epidemic [4].

HIV increases the risk of lymphoma, especially non-Hodgkin’s lymphoma (NHL) [5] resulting in it being categorized as an AIDS defining illness [6]. In Uganda the frequency of lymphomas particularly NHL, the more frequent type has increased since the beginning of the HIV/AIDS pandemic in the early 1980s [7]. The incidence of NHL among HIV infected individuals is 7.3 cases per 100,000, being 6.7-fold higher compared to HIV uninfected persons [9]. The Kampala Cancer Registry reports the incidence of NHL in Kampala is increasing by 6.7% annually in men and 11% annually in women since the beginning of the HIV pandemic.

A study done in Uganda showed that all HIV-positive Ugandans with NHL not receiving HAART die within 1 year of NHL diagnosis [9]. NHL and other AIDS related lymphomas are typically more advanced at presentation [5]. The advanced stage III and stage IV lymphoma disease have been used in the international prognostic index (IPI) tool to predict survival in aggressive lymphoma in which are associated with poor survival rates. It is therefore paramount to have an early diagnosis and treatment of lymphoma before it presents with advanced stage III and IV disease. These diseases have nonspecific symptoms; commonly presenting with lymphadenopathy and constitutional symptoms of fever, weight loss and night sweats, and can also affect extra-nodal sites, giving rise to atypical presentations [9]. These symptoms mimic other HIV opportunistic infections like tuberculosis (TB) [10]. TB is the leading cause of morbidity and mortality in HIV with extra pulmonary TB (EPTB) on a rise in sub-Saharan Africa. TB affects any part of the body resulting in a nonspecific clinical presentation depending on the organ of the body affected including fevers, night sweats, weight loss and lymphadenopathy.

Lymphoma diagnosis is challenging even for pathologists in developed nations without hematopathological specialization [9]. The gold standard for the diagnosis of lymphoma is biopsy and histology. These are invasive and limited by the need for skill and unavailability of pathology centers in many health centers. As a result diagnosis is missed and commonly HIV patients with lymphadenopathy and constitutional symptoms are usually empirically treated for TB resulting into delayed diagnosis of lymphoma [9] especially in rural settings. In a south African retrospective study, 18/21 patients with lymphoma had received anti-TB treatment in the preceding 12 months before diagnosis of lymphoma was made [9]. This study had a small sample size limiting the interpretation of the findings and it did not look at the factors associated with the misdiagnosis. In this study, we aimed to investigate the frequency and factors associated with lymphoma misdiagnosis as TB among a substantial population of HIV positive patients diagnosed with lymphoma attending the Uganda Cancer Institute, Mulago Hospital, Kampala.

## Methods

### Design and setting

**Fig. 1:**
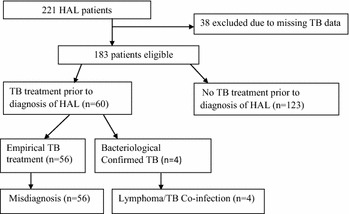
Study inclusion criteria. *TB* tuberculosis, *HAL* HIV associated lymphoma

A retrospective cohort chart review was carried out between February and March 2015 at the Uganda Cancer Institute (UCI) in Kampala located in Mulago Hospital complex. UCI is a national cancer referral center receiving about 3000 new cancer patients annually. Majority of these patients are referred from Mulago National Referral and Teaching Hospital with a histological diagnosis which is then re-assessed at the UCI and biopsy samples reexamined. The Institute receives about 3–4 new patients with lymphoma every month according to local data. Charts of documented HIV infected patients who attended the UCI from January 2004 to November 2014 with a histological diagnosis of lymphoma were reviewed. Patients were allocated into two arms, those with and without a history of TB treatment (Fig. 1). Patients charts who had a history of TB treatment more than 12 months prior to the diagnosis of lymphoma together with patients charts that had missing data on the history of TB treatment were excluded from the study.

Trained data clerks retrieved patient charts from the records department at UCI and extracted the required information into a standard data form. Blood investigations, (Hemoglobin, lactate dehydrogenase and CD4 cell count) and imaging (abdominal ultrasound and chest x-ray) were extracted. The age, sex, address, level of education, clinical presentation of fevers, night sweats, lymphadenopathy and ascites were captured.

History of TB treatment was recorded within the last 12 months prior to diagnosis of lymphoma, site of TB, duration on anti-TB’ treatment before diagnosis of lymphoma and the method of TB diagnosis to capture bacteriological versus empirical diagnosis of TB. Patients with a history of empirical TB treatment with no clinical improvement who later got a histological diagnosis of lymphoma were considered a possible misdiagnosis (Fig. 1). Type and stage of HIV associated lymphoma were also recorded.

### Statistical analysis

Data obtained was checked and entered using EPI-DATA. Analysis was performed using Stata version 13 Software. Frequency of TB misdiagnosis in patients with HIV associated lymphoma was established as a proportion among the total HIV associated lymphoma patients studied. Categorical variables were summarized as percentages and frequencies while continuous variables were summarized as median and interquartile range. To establish factors associated with misdiagnosis of TB in patients with lymphoma, bivariate analysis was performed using a Chi square test statistic for the categorical variables and a Wilcoxon rank-sum (Mann–Whitney) test for the numeric/continuous variables and those with a conservatively set *p* value <0.2 were analyzed using multivariate regression analysis. Variables with final p values <0.05 were considered to be associated with a misdiagnosis of HIV associated lymphoma.

## Results

### Patient characteristics

From February to March 2015; 221 clinical charts of HIV- infected patients with lymphoma attending the Uganda Cancer Institute were reviewed of which 38 were excluded because of missing data on TB treatment leaving a total of 183 patient files.

There were 106/183 (57.9%) males; the median age was 35 years (IQR 28–45). Many of these participants had attained primary education 167/183 (91%). 91/183 (49.7%) were married and 19/183 (10.4%) were widowed. Lymphadenopathy was the commonest presentation at 161/183 (91%) with chest pain at 44/183 (24.3%) and dyspnea at 43/183 (23.5%) being the least. The median CD4 cell count was relatively low, 232 cells/μl. Those who had received anti-TB were treated for a median time of 3.5 (1–5.3) months before a histological diagnosis of lymphoma was made due to failure to respond to TB medication (Table 1). Of the 183 patients with lymphoma, NHL accounted for 119/183 (65%) and Hodgkin’s lymphoma (HL) was 64/183 (30%). Most patients 142/183 (77.6%) presented with advanced cancer, with mainly stage III and IV lymphoma, 7/183 (3.8%) had stage I lymphoma disease. Only 120 out of 183 (65.4%) patients were on highly active anti-retroviral therapy.

**Table 1:** Baseline Characteristics of HIV/lymphoma patients

Baseline characteristics	n	%
Gender (n = 183)		
Female	77	42.1
Male	106	57.9
Marital status (n = 183)		
Divorced/separated	36	19.7
Married	91	49.7
Single	37	20.2
Widowed	19	10.4
Occupation (n = 183)		
Casual laborer	39	21.3
Civil servant	30	16.4
House wife	33	18
Peasant	41	22.4
Trader	40	21.9
Education (n = 183)		
None	16	8.7
Primary	80	43.7
Secondary	63	34.4
Tertiary	24	13.1
Fever (n = 183)		
No	37	20.2
Yes	146	79.8
Chest pain (n = 181)		
No	137	75.7
Yes	44	24.3
Cough (n = 183)		
No	116	63.4
Yes	67	36.6
Drenching night sweats (n = 183)		
No	36	19.7
Yes	147	80.3
Loss of appetite (n = 180)		
No	37	20.6
Yes	143	79.4
Lymph node swelling (n = 177)		
No	16	9
Yes	161	91
Dyspnea (n = 183)		
No	140	76.5
Yes	43	23.5

	Median (IQR)
Age (years)	35 (28–45)
HAART duration (months)	18 (5–18)
Duration on anti TB (months)	3.5 (1–5.3)
CD4 count	232 (124–387)
Body mass index	19.5 (17.1–21.6)
Hemoglobin	10.2 (8.4–12.1)
LDH value	462 (302–772)

*HAART* anti-retroviral therapy, *LDH* lactate dehydrogenase, *IQR* inter quartile range

Sixty patients (32.8%) had history of TB treatment of which 56/183 (30.6%) had received empirical TB treatment while 4/183 (0.02%) had treatment for bacteriologically confirmed TB. Majority of the patients with a history of TB treatment 51/60 (85%) had been treated for EPTB. The 56 patients [56/183 (30.6%)], with initial empirical TB treatment did not improve with TB treatment, and were considered to have a possible misdiagnosis (Fig. 1; Table 2).

**Table 2:** Clinical characteristics of HIV associated lymphoma patients

Lymphoma type, ART and TB status	n	%
Documented lymphoma type		
HL	64	35
NHL	119	65
Total	183	100.0
HAART		
No	63	34.4
Yes	120	65.4
Total	183	100.0
Lymphoma stage		
Stage I	7	3.8
Stage II	34	18.6
Stage III	71	38.8
Stage IV	71	38.8
Total	183	100.0
Site of TB		
EPTB	51	85.0
PTB	9	15.0
Total	60	100.0

*HAART* anti-retroviral therapy, *TB* tuberculosis, *HL* Hodgkin’s lymphoma, *NHL* non Hodgkin’s lymphoma, *EPTB* extra pulmonary tuberculosis, *PTB* pulmonary tuberculosis

### Comparison of characteristics between possible misdiagnosis and definite diagnosis

Table 3 shows the results of the bivariate analysis in which stage of lymphoma [OR 4.03 (95% CI 1.49–10.93, p < 0.006)], dyspnea [OR 2.46 (95% CI 1.01–5.97, p < 0.01)], fever [OR 3.62 (95% CI 1.31–10.01, p < 0.016)], drenching night sweats [OR 4.41 (95% CI 1.45–3.37, p < 0.008)], chest pain [OR 3.3 (95% CI 1.46–7.46, p < 0.004)], hemoglobin level [OR 0.88 (95% CI 0.78–0.99, p < 0.034)], ART status [OR 1.88 (95% CI 0.93–3.8, p < 0.070)] and loss of appetite [OR 4.17 (95% CI 1.51–11.52, p < 0.016)], were associated with possible lymphoma misdiagnosis as TB.

**Table 3:** Comparison of characteristics between possible misdiagnosis and definitive diagnosis

Characteristics	No misdiagnosis n (%)	Misdiagnosis n (%)	P-value
Fever			
No	32 (86.5)	5 (13.5)	
Yes	95 (65.1)	51 (34.9)	*0.012*
Chest pain			
No	107 (78.1)	30 (21.9)	
Yes	18 (40.9)	26 (59.1)	*0.001*
Drenching night sweats			
No	32 (88.9)	4 (11.1)	
Yes	95 (64.6)	52 (35.4)	*0.005*
Loss of appetite			
No	32 (86.5)	5 (13.5)	
Yes	93 (65.0)	50 (35.0)	*0.012*
Dyspnea			
No	104 (74.3)	36 (25.7)	
Yes	23 (53.5)	20 (46.5)	*0.010*
HAART status			
No	49 (77.8)	14 (22.2)	
Yes	78 (65.0)	42 (35.0)	*0.075*
Stage of lymphoma			
Stage I and II	36 (87.8)	5 (12.2)	
Stage III and IV	91 (64.1)	51 (35.9)	*0.004*
Cough			
No	84 (72.4)	32 (27.6)	
Yes	43 (64.2)	24 (35.2)	0.240
Lymph node swelling			
No	12 (75.0)	4 (25.0)	
Yes	113 (70.9)	48 (29.8)	0.680

	Median (IQR)	Median (IQR)	
HAART duration (months) duration (months) duration (months)	24 (8–36)	12 (4–36)	*0.020*
CD4 count	275 (146–458)	172 (96–234)	*0.001*
Hemoglobin	10.5 (9–12.4)	9.8 (7.1–11.3)	*0.020*
LDH value	462 (319–721)	468 (263–845)	0.880

No association with Gender, Marital status, Education and Body mass index

*HAART* anti-retroviral therapy, *LDH* lactate dehydrogenase, IQR inter quartile range

These variables were further used for multivariate analysis to determine the factors associated with possible misdiagnosis of HIV/lymphoma co infected patients as TB patients. The multivariate analysis shown in Table 4 revealed chest pain and stage of lymphoma to be independently associated with possible misdiagnosis of lymphoma aOR 1.46 (95% CI 0.41–5.23, p < 0.001) and aOR 3.22 (95% CI 1.08–9.63, p < 0.04) respectively. The CD4 count was excluded at multivariate analysis due to missing data. ART duration was excluded because not every study participant was on HAART.

**Table 4:** Multivariate analysis for possible misdiagnosis of HIV associated lymphoma patients

Characteristics	Crude odds	P-value	Adjusted odds	P-value
Chest pain				
No	1.00		1.00	
Yes	3.3 (1.46–7.46)	*0.004*	4.4 (1.89–10.58)	*0.001*
Stage of lymphoma				
Stage I and II	1.00		1.00	
Stage III and IV	4.03 (1.49–10.93)	*0.006*	3.22 (1.08–9.63)	*0.037*
Drenching night sweats				
No	1.00		1.00	
Yes	4.41 (1.45–3.37)	0.008	1.44 (0.39–5.24)	0.585
Loss of appetite				
No	1.00		1.00	
Yes	4.17 (1.51–11.52)	0.016	2.85 (0.76–10.77)	0.122
Dyspnea				
No	1.00		1.00	
Yes	2.46 (1.01–5.97)	0.010	1.31 (0.56–3.04)	0.535
Highly Active Anti-retroviral Therapy status				
No	1.00		1.00	
Yes	1.88 (0.93–3.8)	0.070	1.82 (0.82–4.09)	0.142
Fever				
No	1.00		1.00	
Yes	3.62 (1.31–10.01)	0.016	1.46 (0.41–5.23)	0.563
Hemoglobin	0.88 (0.78–0.99)	0.034	0.94 (0.82–1.07)	0.344

## Discussion

In our study we found three in every ten patients with HIV associated lymphoma attending UCI, had a possible misdiagnosis before a final diagnosis of lymphoma was made. These patients were treated for TB for a median period of 16 weeks prior to correct diagnosis. The WHO 2003 TB treatment guidelines for national programmes suggest empirical TB treatment in HIV infected patients with symptoms of TB, stressing the importance of follow-up investigations and reviews of treatment response after 1 month [11] to rule out other diseases that mimic TB. If followed as recommended, early review of treatment response offers opportunities for earlier investigation and diagnosis of other diseases especially lymphoma that mimics TB in order to improve survival.

Majority of these patients were treated for EPTB, and therefore stand a high chance to be registered as EPTB treatment failures in national statistics and for this reason, this high frequency of misdiagnosis of lymphoma as TB could be contributing to the low EPTB treatment success rate of 66% reported by the WHO for Uganda [11].

Our study is the first to explore clinical symptoms and signs that are associated with possible misdiagnosis of TB among lymphoma patients. We found all patients in our retrospective cohort to be symptomatic with more than one constitutional symptom. Chest pain, stage of lymphoma, fever, HAART status, low hemoglobin, dyspnea, drenching night sweats and loss of appetite were the main factors found to be associated with TB misdiagnosis in bivariate analysis. Although many patients presented with lymphadenopathy, it was not found to be associated with misdiagnosis of Lymphoma. The risk of being initiated on empirical anti-TB treatment prior to the diagnosis of lymphoma was 4.4 times in cases where one had chest pain and 3.2 times in cases where one had stage III and IV lymphoma disease as compared to no chest pain and stage I and II lymphoma disease respectively. This indicates that advanced lymphoma (stage III and IV) is symptomatically very similar to TB as chest pain is a common symptom in TB [9].

Our findings were somewhat different from the South African study with a lower frequency of misdiagnosis 30.6% compared to theirs, 85.7% and shorter length of TB treatment prior to lymphoma diagnosis. These differences may be explained by a smaller sample size in the South African study of (21 patients) or the retrospective nature of the South African study although similar to ours, dealt with patients directly rather than data.

Worryingly our data also showed a low rate of ART (65.4%). Our study did not allow for CD4 count analysis as it was not undertaken in the HIV clinic setting, however, a lymphoma diagnosis gives a patient an HIV WHO stage of IV which necessitates ART initiation. Earlier work in Uganda shows that mortality in those with lymphoma and HIV not on ART is 100%. HIV and cancer specialists should consider strategies that increase communication for patient care, such as the integrated HIV–TB model that has streamlined care for co-infected patients and decreased mortality.

Our retrospective design and tertiary care setting, would have led to an underestimation of the true rate of misdiagnosis as some patients may have died or been lost to follow-up before being referred to the UCI. Data was collected using non-standardized clinical notes extracted from medical charts which potentially lead to recording bias, selection bias and missing data. Despite these limitations, we feel that our findings send an important message to clinicians managing patients empirically treated with TB, especially EPTB to be alert from the beginning to the possibility of an alternative diagnosis of HIV associated lymphoma.

This study showed a co-morbidity rate of lymphoma and TB to be 6.7%, which is consistent with a study by De La Rosa et al. [12] that showed a co-occurrence of 6%. Therefore, clinicians have to consider an additional as well as alternative diagnosis if the response to either lymphoma or TB treatment is not achieved.

## Conclusion

A high proportion of patients with HIV associated lymphoma attending UCI are possibly misdiagnosed and treated as TB. Chest pain and stage III and IV of lymphoma were associated with a risk of possible misdiagnosis of lymphoma as TB. Clinicians treating HIV patients for TB should have a low threshold for investigating lymphoma.
